# Beta-glucan contamination of pharmaceutical products: How much should we accept?

**DOI:** 10.1007/s00262-016-1875-9

**Published:** 2016-07-29

**Authors:** Claire Barton, Kim Vigor, Robert Scott, Paul Jones, Heike Lentfer, Heather J. Bax, Debra H. Josephs, Sophia N. Karagiannis, James F. Spicer

**Affiliations:** 1 Cancer Research UK Centre for Drug Development, Cancer Research UK, Angel Building, 407 St John Street, London, EC1V 4AD UK; 2 Biotherapeutics Development Unit, Cancer Research UK, South Mimms, Hertfordshire EN6 3LD UK; 3 Division of Genetics and Molecular Medicine, Faculty of Life Sciences and Medicine, St. John’s Institute of Dermatology, King’s College London, 9th Floor, Guy’s Tower Wing, Guy’s Hospital, London, SE1 9RT UK; 4 NIHR Biomedical Research Centre at Guy’s and St. Thomas’s Hospitals, King’s College London, 9th Floor, Guy’s Tower Wing, Guy’s Hospital, London, SE1 9RT UK; 5 Division of Cancer Studies, Department of Research Oncology, Faculty of Life Sciences and Medicine, King’s College London, 3rd Floor Bermondsey Wing, Guy’s Hospital, Great Maze Pond, London, SE1 9RT UK

**Keywords:** Beta-glucan, Lentinan, Biotherapeutics, Antibodies, Cancer, Immunostimulation

## Abstract

Beta-glucans are large polysaccharides produced by a range of prokaryotic and eukaryotic organisms. They have potential immunostimulatory properties and have been used with therapeutic intent as anti-microbial and anti-tumour agents. A range of other potentially beneficial effects have been described, and oral forms of beta-glucans are widely available over-the-counter and online. Parenteral formulations are popular in parts of Asia and are the subject of ongoing trials, worldwide. Beta-glucans are also potential contaminants of pharmaceutical products, and high levels have been described in some blood products. However, little is known about the clinical effects of such contamination, considerable uncertainty exists over the level at which immunostimulation may occur, and there are no guidelines available on acceptable levels. We encountered beta-glucan contamination of one of our products, and we suspect that others may encounter similar issues since the origin of beta-glucan contamination includes commonly used filters and solutions applied in the manufacture of biotherapeutic agents. It is likely that regulators will increasingly enquire about beta-glucan levels in pharmaceutical products, especially those with an immunomodulatory mechanism of action. Here, we review the literature on beta-glucans in pharmaceutical products and propose an acceptable level for therapeutic agents for parenteral use.

## Introduction

**Fig. 1:**
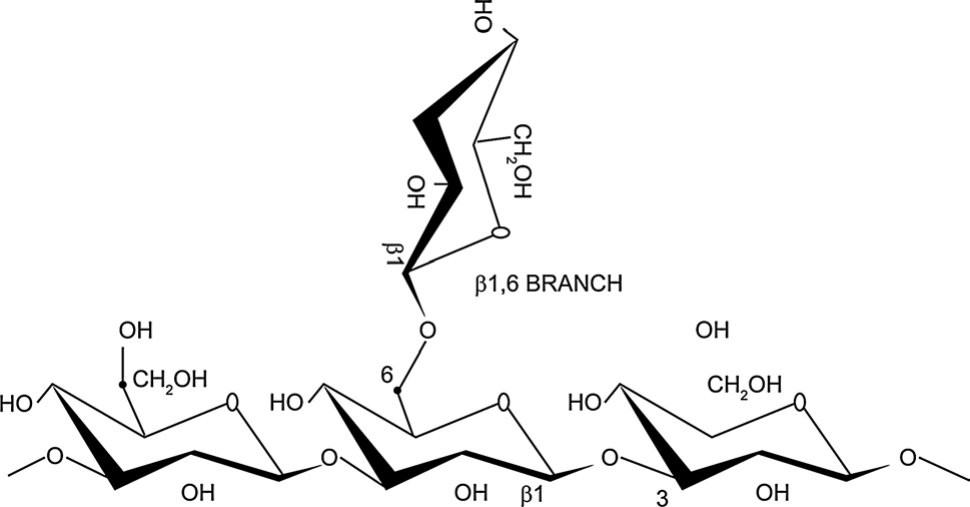
Beta-glucans are polysaccharides composed of d-glucose monomers linked by (1–3) beta-glycosidic bonds. A simple linear 1,3 glycosidic chain with a single 1,6 glycosidic branch is illustrated here, but there are many variations (figure derived from Chan et al. [23], originally published in BioMed Central)

Beta-glucans are polysaccharides of d-glucose monomers linked by (1–3) beta-glycosidic bonds (see Fig. 1 for an example). They are structurally diverse, and differences in the length and branching of side chains result in differences in solubility and biological activity (reviewed [1]). Beta-glucans are found in the cell walls of a wide range of prokaryotic and eukaryotic organisms, including yeast, fungi, seaweeds and cereals. They are a potential contaminant in pharmaceutical products, originating from cellulose-based filters and other raw materials used in pharmaceutical processing. Beta-glucans are similar to endotoxins in that they are large polysaccharides that can elicit an inflammatory response. Currently, endotoxins are strictly regulated in terms of maximum permitted levels in pharmaceutical products (ICH Q6A and Q6B Specifications, 1999 [2, 3]). Specifications are also in place for residual proteins and polypeptides in biological products (ICH Q6B Specifications, 1999 [3]), but there is no such guidance available for beta-glucans [4].

Recently we encountered contamination of a biotherapeutic agent by beta-glucans. The product, MOv18 IgE (an IgE directed towards the tumour-associated antigen, folate receptor-alpha), was manufactured at the Cancer Research UK, Biotherapeutics Unit for a phase I clinical trial (CRUKD/14/001; EudraCT number 2014-000070-19) in patients with advanced malignancies, in the United Kingdom (UK). This is the first trial in which a monoclonal IgE is administered to patients with therapeutic intent.

Extensive non-clinical testing indicates that anaphylactic reactions should not occur with MOv18 IgE [5], and all patients in the trial are scheduled to undergo intradermal testing prior to every infusion to avoid administration of MOv18 IgE to any patient at risk of an anaphylactic reaction due to MOv18 IgE itself or to any other constituent. However, infusion-related reactions due to non-specific cytokine release could occur with MOv18 IgE, as commonly seen with therapeutic IgG antibodies and other biological agents. At the time of occurrence, such reactions could be confused with anaphylaxis. However, evaluation of serum tryptase levels should enable infusion-related reactions due to non-specific cytokine release to be distinguished retrospectively from anaphylaxis due to mast cell or basophil degranulation triggered by MOv18 IgE. Since infusion-type reactions have been reported with rapid intravenous (i.v.) administration of beta-glucans [6, 7], it was considered particularly important to ensure that levels of beta-glucans in the MOv18 IgE preparation were sufficiently low for there to be no adverse reactions attributable to co-administration of beta-glucans in the clinical trial. Beta-glucans have also been reported to have immunostimulatory properties when administered orally or parenterally, and we also wished to avoid any immunostimulatory effect from co-administered beta-glucans which could cause confusion with the anticipated immunologically mediated anti-tumour activity of MOv18 IgE [8–12].

The source of contamination of MOv18 IgE was extensively investigated and turned out to be commonly used cellulose filters and sucrose-containing solutions. Measures were taken to reduce the levels of contamination through additional downstream processing and extra washing steps. This led to an almost 10-fold reduction in beta-glucan levels, which now lie within the range found in commercially available antibodies used in oncology [13]. Subsequent batches of MOv18 IgE contained around 3 ng/mg of beta-glucans. Based on levels of beta-glucans detected in the bulk drug substance from development, scale up and clinical batches of MOv18 IgE, and a review of the literature on the possible clinical implications of beta-glucans administration, we submitted an amendment to the Investigational Medicinal Product Dossier for our product to include a specification of 10 ng/mg for beta-glucan contamination. With this level of contamination, we estimated that up to 500 ng of beta-glucans could potentially be delivered on a single occasion to an individual patient in the trial. A 500 ng dose could result in plasma levels of around 100 pg/mL assuming distribution only to the circulating volume and no clearance. This specification was accepted by the UK Medicines and Healthcare Products Regulatory Agency. From our review of the literature, this appears to be the first time a specification limit for beta-glucans, approved by a regulatory authority, has been reported.

With cellulose-based filters and sucrose-based formulation buffers commonly used in the manufacture of biotherapeutic products, we suspect that other scientists and clinicians working on the research and development of biotherapeutics for use in patients with cancer and other diseases will encounter beta-glucan contamination. Furthermore, regulatory authorities are now more likely to enquire about beta-glucans in biotherapeutic products. This issue may be particularly important in oncology with the advent of new therapeutics, including monoclonal antibodies and other biological agents, which have a variety of complex immunomodulatory mechanisms of action.

The measures taken to identify the source of contamination and to reduce the levels of beta-glucans contamination in our product are described in detail in a separate publication [13]. Here, we provide an overview of the safety and tolerability of beta-glucans as contaminants of other therapeutic agents, and as a therapeutic agent in its own right, based on a review of the literature. After a brief review of insoluble/particulate beta-glucans (generally orally administered), this overview concentrates on soluble forms since these are most relevant to contamination of biotherapeutic agents, such as monoclonal antibodies and their derivatives, which are given parenterally.

## Insoluble (particulate) beta-glucans

Oral forms of beta-glucans are widely available over-the-counter as oat bran and bran-based products such as breakfast cereals, cereal bars and drinks. These are promoted mainly for their clinically proven cholesterol-lowering properties ([14] and reviewed in [15]), which are complemented by favourable effects on glucose metabolism [16] and possibly other cardiovascular risk factors (reviewed in [17]). There is also evidence that orally administered beta-glucans have beneficial anti-inflammatory and pro-apoptotic effects in inflammatory bowel disease and colitis-associated colon cancer [18].

Beta-glucan supplements are also readily available online as tablets, capsules and other oral formulations (see for example, Amazon [19] or the Web MD website [20]). These forms of beta-glucans are more frequently derived from sources other than oat bran (such as yeast or fungi) and tend to be promoted as immune stimulants, protecting against microbial infections and cancer.

Topical beta-glucans are also used in cosmetics and as “cosmeceuticals”, for improving skin health and appearance, based on non-clinical studies demonstrating beneficial effects of beta-glucans on wound healing, inflammation, ageing (antioxidant and anti-wrinkle activity) and moisturisation (reviewed in [21]).

## Immunological effects of beta-glucans

### Effects on host immune defence

The immunomodulatory properties of beta-glucans have been the subject of considerable scientific study in the Western world for several decades. Non-clinical studies indicate that beta-glucans activate innate immunity as well as adaptive immunity, modulating both humoral and cell-mediated immune responses. Beta-glucans act on several immune receptors including Dectin-1 (considered the main receptor for beta-glucans [22]), complement receptor-3 (CR3) and toll-like receptors 2 and 6 (TLR-2/6) (reviewed in [23–26]). Beta-glucans have been found to increase the anti-microbial activity of mononuclear cells and neutrophils, to enhance the functional activity of macrophages, to stimulate the proliferation of monocytes and macrophages and to stimulate the production of proinflammatory molecules such as complement components, interleukin (IL)-1α/β, TNF-α, IL-2, interferon (IFN)-γ, eicosanoids, IL-4 and IL-10. Consistent with these findings, beta-glucans have also been shown to protect against or ameliorate the effects of bacterial and other infections in animal models (reviewed in [23–26]). The broad-ranging immunological effects of beta-glucans and the epidemiological associations between environmental beta-glucans exposure and respiratory allergies have also led to interest in their use in the prevention or treatment of allergic diseases. The ability of beta-glucans to rebalance a dysregulated Th1/Th2 equilibrium is thought to be beneficial in these conditions (reviewed in [27]).

It is thought that the immunological effects of orally administered beta-glucans are essentially the same as those of parenterally administered beta-glucans. Orally administered beta-glucans are absorbed through the gastrointestinal tract and taken up by tissue-resident macrophages. Here, they are fragmented, transported to the bone marrow and reticuloendothelial system and eventually released and taken up by other immune cells, leading to various immunological effects (reviewed in [23]). On the basis of these features, particulate beta-glucans have been evaluated as potential vaccines for invasive fungal diseases (reviewed in [28]) and beta-glucans particles have been proposed as a delivery system for oral vaccines, acting as both carrier and adjuvant [29].

### Potential anti-cancer effects

It has been suggested that beta-glucans, administered alone, in combination with monoclonal antibodies, or as adjuvants alongside vaccines or other types of immunotherapy, could help reduce cancer growth. Beta-glucans may act either within tumour microenvironments (TMEs) or systemically, by activating or recruiting immune effector cells into tumours or by augmenting adaptive immune responses triggered by concurrent immunotherapy.

Beta-glucan administration has been reported to influence polarisation of tumour-associated macrophages away from an immunosuppressive and towards an activatory and tumouricidal phenotype (M1). This polarisation is thought to be mediated by engaging the cell surface C-type lectin receptor Dectin-1 and triggering the spleen tyrosine kinase (Syk)-Card9-Erk pathway via the ITAM domain [30]. This may result in production of inflammatory cytokines by macrophages and antigen-presenting cells, and tumour growth restriction through enhanced phagocytosis, antigen presentation and induction of Th1 and CTL responses. In one study, beta-glucan administration to mammary tumour-bearing mice triggered IL-12 production by macrophages which led to a switch from IL-4- to IFN-γ-producing Th1 cells [31]. Another study reported reversal of MDSC immunosuppressive phenotypes in response to particulate beta-glucan treatment. The beta-glucan promoted mature CD11c^+^ F4/80^+^ Ly6C^low^ MDSCs via a Dectin-1/NF-κB-dependent pathway, and this was associated with enhanced infiltration of dendritic cells, macrophages and CTLs in tumours [32].

Well-known immunosuppressive forces in TMEs can profoundly impair the maturation and antigen-presenting functions of DCs. TME-associated inflammation promotes IL-10- and TGFβ- secreting DC phenotypes, which supports accumulation of regulatory T cells. These effects have been shown to be reversed upon administration of beta-glucans and are accompanied by a reduction in regulatory T cells and enhanced tumour infiltration by mature DCs, macrophages and granulocytes in mouse models of cancer [33, 34]. Early data suggest potential enhancement of PD-L1 expression on mouse peritoneal macrophages by microparticulate beta-glucan, which could have a negative impact on T cell survival and activation [35]. However, potential effects in relation to cancer-associated immune surveillance, immunomodulation or treatment with anti-PD-1 checkpoint inhibitors are yet to be explored.

The potential administration of beta-glucans in combination with antibodies, vaccines and other immunotherapeutic or chemotherapeutic agents has been investigated in pre-clinical studies. Monoclonal antibodies, especially those which function via complement activation, have shown improved efficacy in animal models of cancer when co-administered with beta-glucans. These effects, most likely due to the ability of beta-glucans to bind complement receptor-3 (CR3) and to promote antibody-mediated complement activation against cancer cells, were also associated with recruitment of tumouricidal granulocytes to tumour sites [36, 37]. Consistent with these findings, encouraging efficacy was reported in a non-randomised phase II clinical study, in which the anti-EGFR complement-activating antibody cetuximab was administered in combination with a soluble β-1,3/1,6-glucan to patients with KRAS-mutant/EGFR signalling-resistant colorectal carcinomas [38]. Results of phase I/II trials of beta-glucans in combination with other immunotherapeutic agents also indicate good tolerability with promising signs of anti-tumour activity in patients with chronic lymphocytic leukaemia and neuroblastoma [39, 40].

### Dose–response considerations

Generally, non-clinical and clinical studies of the immunological effects of beta-glucans have not been designed to establish a dose–response relationship between beta-glucans and the effect of interest at low doses/concentrations, or to define the lowest dose/concentration at which immunological effects occur. However, based on a range of nonclinical studies, it seems unlikely that clinically significant immunological effects would occur with serum levels of 100 pg/mL or lower. One in vitro study showed that exposure to 100 pg/mL of beta-glucans for 48 h stimulated the production of IL-17 by dendritic cells in mixed lymphocyte reaction assays, but stimulation was most marked at 1 ng/mL (10 times higher than 100 pg/mL) and there was no effect on IFN-γ or IL-5 production at concentrations up to 100 µg/mL [41]. In a separate study, some induction of IL-1β and IL-12 by macrophages was detected at beta-glucans concentrations as low as 10 ng/mL (100 times higher than 100 pg/mL) [42]. However, production of these cytokines was much greater at higher concentrations, and for macrophages to produce TNF-α or IFN-γ, a beta-glucans concentration of at least 0.1 µg/mL (100 ng/mL) was required. In another study, an enhanced oxidative burst response and microbial killing by peripheral blood mononuclear cells were detected at beta-glucans levels of 100 ng/mL (0.1 μg/mL) or greater, but stimulation of the NF-κB-like DNA-binding protein by beta-glucans only occurred at concentrations of 370 ng/mL (0.37 μg/mL) and above [43].

A range of immunological effects have been demonstrated with beta-glucans concentrations between 0.2 and 10 µg/mL, including dendritic cell activation and maturation [44–46], neutrophil chemotaxis [47], histamine release from basophils [48] and tumour cell killing by polymorphonuclear leucocytes [49]. For example, in one study, as little as 3 µg/mL of beta-glucans induced the production of intracellular and membrane-associated IL-1, but induction of secreted IL-1 required 25 µg/mL, with 50 µg/mL yielding maximal responses. Production of prostaglandin-E2 (PGE2) by glucan-activated human monocytes occurred at concentrations of as low as 12.5 µg/mL but plateaued at 25 µg/mL [45]. Examples of in vitro studies in which the immunological effects of beta-glucans have been evaluated at low concentrations are summarised in Table 1. Of note, many of the studies which looked for a dose–response effect also looked at duration of exposure. To see immunological effects at lower concentrations, relatively prolonged exposure to beta-glucans (12–72 h) may have been required.

**Table 1:** Examples of in vitro studies of the immunological effects of soluble beta-glucans in which low concentrations have been evaluated

References	Immunological effect studied	Type of beta-glucans studied and context	Beta-glucan concentrations evaluated	Lowest beta-glucan concentration at which any effects were detected
[41]	Mixed lymphocyte reactions between monocyte-derived dendritic cells and allogeneic naïve CD4+ T cells Expression of Jagged1 mRNA	Curdlan (beta-glucan derived from C. albicans) Moncyte-derived dendritic cells and T cells from PBMCs from healthy donors Phorbol myristate acetate (PMA)-differentiated THP-1 macrophages (derived from a leukaemia cell line)	0.1–100 ng/mL (for 48 h)	0.1 ng/mL of curdlan led to production of IL-17 by dendritic cells in mixed lymphocyte reaction assays (production most marked at 1 ng/mL), but curdlan had no effect on IFN-ɣ or IL-5 production 10–100 ng/mL (but not 1 ng/mL) increased Jagged1 mRNA expression in dendritic cells, but a concentration of 10 µg/mL was needed in PMA-stimulated THP-1 cells (macrophages derived from a human monocytic leukaemia cell line)
[42]	Splenocyte proliferation Macrophage cytokine production (IL-1β, IFN-ɣ, IL-12) Macrophage and NK cell tumouricidal activity	Beta-glucans from mutated and wild-type S. cerevisiae (yeast) (beta-glucans from mutant yeast is known to contain more mannose than beta-glucans from wild-type yeast) Mouse splenocytes and peritoneal macrophages	0.01–100 µg/mL	0.01 µg/mL (= 10 ng/mL) of mutant (but not wild-type) beta-glucans induced detectable splenocyte proliferation and IL-1β and IL-12 production from macrophages; higher concentrations needed for TNFα and IFN-ɣ induction. Higher concentrations also needed for induction of any cytokine or splenocyte proliferation by wild-type beta-glucan
[43]	Cytokine release Oxidative burst response Microbicidal activity Activation of a NF-kB-like factor	Yeast-derived soluble beta-glucan (PGG glucan) Whole blood and purified subpopulations of PBMCs from healthy volunteers	0.1–100 µg/mL	0.1 µg/mL (= 100 ng/mL) enhanced the leucocyte oxidative burst response and microbial killing of whole blood, but concentrations up to 100 µg/mL did not result in cytokine release from PBMCs (IL-1α, IL-1β, IL-6, IL-8 and TNF-α); activation of an NF-kB-like factor was detectable at 0.37 µg/mL and above
[44]	Activation and maturation of immature dendritic cells measured by Cell surface expression of CD80, CD86, CD83, CD40, CD54 and HLA-DR Production of interleukin IL-12 and IL-10 (proteins and mRNA expression) Capacity for endocytosis (a marker of immaturity) Ability to cause T cell stimulation (as measured by T cell secretion of IFN-ɣ and IL-10 after co-incubation) IκB and p38 MAPK phosphorylation,and NF- κB translocation	Beta-glucans from Ganoderma lucidum (Lingzhi or Reishi mushrooms used in Chinese/Japanese herbal medicines) Human monocyte-derived dendritic cells and T cells from healthy donors	0.2–200 µg/mL	Concentrations of beta-glucans as low as 0.2 µg/mL (200 ng/mL) enhanced production of IL-12 p40, and IL-10 from dendritic cells (lower concentrations not tested); much higher cytokine production was stimulated by 10 µg/mL 10 µg/mL induced maturation of dendritic cells (assessed by cell membrane markers) (lower concentrations not tested) Endocytic activity of dendritic cells was suppressed by 10 µg/mL for 24 h (other concentrations not tested) After exposure to 10 µg/mL for 24 h, dendritic cells enhanced T cell proliferation and activation, as evidenced by T cell secretion of IFN-ɣ and IL-10 (other concentrations not tested) 10 µg/mL for 30 min induced MAPK phosphorylation; 10 µg/mL for 60 min induced IκB phosphorylation; and 10 µg/mL for 2 h induced NFKB translocation to the nucleus (all indicators of dendritic cell maturation) (lower concentrations not tested)
[47]	Neutrophil migration/chemotaxis	Highly purified beta-glucans of different structures, derived from different sources, including yeast (Candida albicans-derived beta-glucans), fungi (including lentinan, grifolan and sonifilan), bacteria (curdlan) and algae (laminarin) Human neutrophils from healthy donors	0.1–200 μg/mL	Concentrations of Candida albicans-derived beta-glucans ≥1 μg/mL significantly enhanced neutrophil migration/chemotaxis (no enhancement seen at 0.1 μg/mL). None of the other beta-glucans, even at 200 μg/mL, enhanced neutrophil migration
[45]	PBMC proliferation Phenotypic and functional maturation of dendritic cells, including IL-12 and IL-10 production	Beta-glucans from mycelium and spores of Ganoderma lucidum, and from barley (different sources and purity) Monocyte-derived dendritic cells and T cells from PBMCs from healthy donors	1–1000 μg/mL	Beta-glucans concentrations between 1 and 10 μg/mL stimulated PBMC proliferation, depending on the source of beta-glucans (a 10-fold difference in potency) 100 μg/mL for 48 h stimulated dendritic cell maturation based on cell surface markers (lower concentrations not tested)
[48]	Histamine release from human blood leucocytes	Curdlan, laminarin, scleroglucan and pustulan (structurally diverse beta-glucans) Leucocytes (including 2 % basophils) from healthy donors and donors allergic to house dust mite	10 ng/mL–100 µg/mL	None of the beta-glucans triggered histamine release at any concentration. However, 1 µg/mL of laminarin, 10 µg/mL of pustulan, 100 µg/mL of curdlan and 300 µg/mL of scleroglucan potentiated anti-IgE antibody-mediated histamine release; 10 µg/mL of laminarin potentiated mite-allergen-induced histamine release in cells from an allergic subject
[46]	IL-1, TNFα and PGE2 production Antigen presentation T4 cell proliferation	Soluble (aminated) beta-glucan of yeast origin Human PBMCs from healthy donors	3–100 µg/mL	3 µg/mL of beta-glucan resulted in intracellular and membrane-associated IL-1 production, but 25 µg/mL was required for secretion of IL-1. PGE2 production was detected at levels as low as 12.5 µg/mL (lower concentrations not tested). TNFα production was detected at 25 µg/mL (lower levels not tested). No effects on antigen presentation or T4 proliferation were detected at levels up to 100 µg/mL
[49]	Cytotoxic activity of polymorphonuclear and other cells against tumour cells	28 immunomodulators, including lentinan and beta-glucans from other sources Mouse macrophages, polymorphonuclear cells, splenocytes and thymus cells 5 tumour cell lines	0.1–100 µg/mL	Lentinan did not induce cytotoxicity at any dose up to 100 µg/mL. However, a linear β-1,3-glucans without branching or carboxymethyl groups was highly effective, inducing ~100 % cytotoxicity at 6.3 µg/mL and above (0.1, 0.4 and 1.6 µg/mL also tested)
[25]	AKT signalling pathway, ERK and c-Raf phosphorylation Cytokine and chemokine secretion (CD54, IL-1α, IL-1β, IL-16, IL-17, IL-23, IFN-γ, CCL1, CCL3, CCL4, CCL12, CXCL10, TIMP-1 and G-CSF) by mouse macrophages Cytokine and chemokine secretion (IL-6, CCL2, CCL3, CCL5, CXCL1 and MIF) by human PBMCs	β-glu6, a synthetic analogue of the lentinan basic unit Mouse peritoneal macrophages and human PBMCs from healthy donors	10–1000 µg/mL	β-glu6 suppressed AKT phosphorylation at 10, 25 and 50 µg/mL (lower concentrations not tested), but suppression only considered significant at 100 µg/mL. Modulation of other AKT pathway components, ERK 1/2 pathway, and cytokine/chemokine production by mouse macrophages and human PBMCs demonstrated at 100 µg/mL (lower concentrations not tested)
[50]	Inflammation-related gene expression kinetics (IL-1, IL-8, NF-kB and IL-10)	Beta-glucans from different sources (oat, barley and shiitake mushrooms) and of different purity/processings PMA-differentiated THP-1 macrophages	100 µg/mL	All tested beta-glucans mildly upregulated the inflammation-related genes with differential gene expression patterns (only 100 µg/mL seems to have been tested). No effect was detected on production of nitric oxide, hydrogen peroxide or phagocytic activity
[51]	Induction of TNFα and IFN-ɣ	Beta-glucans from Ganoderma lucidum PBMCs from a healthy donor	3.25–400 µg/mL	Moderate TNFα induction detected in 5/5 samples at 100 µg/mL or above but in only 2/5 samples below 100 µg/mL. Low level IFN- ɣ induction detected in 2 of 5 samples at 100 µg/mL (but not at 12.5 µg/mL)

*G*-*CSF* granulocyte colony-stimulating factor, *HLA* human leucocyte antigen, *IFN* interferon, *IκB* inhibitor of κB kinase, *IL* interleukin, *MAPK* mitogen-activated protein kinase, *MIF* migration inhibitory factor, *mRNA* messenger ribonucleic acid, *NF*-*κB* nuclear factor-kappa B, *PBMCs* peripheral blood mononuclear cells, *PGE2* prostaglandin E2, *PMA* phorbol myristate acetate, *TIMP* tissue inhibitor of metalloproteinase, and *TNFα* tumour necrosis factor-alpha

Of note, in vitro studies in which the direct anti-tumour effects of beta-glucans have been evaluated suggest that higher concentrations of beta-glucans are required than those needed for immune modulation. For example, in an in vitro cytotoxicity analysis, beta-glucans concentrations up to 100 μg/mL did not directly affect the growth of colon 26-M3.1 cells [42]. In other studies, the proliferation of B16-F10 melanoma cells was reduced by 51 % after 48 h exposure to 750 μg/mL of beta-glucans [52]; proliferation of the gastric cancer cell line SGC-7901 was reduced in a dose-dependent manner at concentrations between 125 and 1000 μg/mL, with around 50 % inhibition with 400 μg/mL [53]; and proliferation of MCF-7 breast cancer cells was reduced in a dose-dependent manner at concentrations between 12.5 and 400 μg/mL, with 50 % inhibition at 400 μg/mL [54].

## Non-clinical safety data for parenteral beta-glucans

Most publications in the English/Western literature which describe administration of beta-glucans with therapeutic intent for malignant or other diseases (notably human immunodeficiency virus [HIV] infections) involve oral administration of beta-glucans. However, in the 1980s and more recently, soluble forms of beta-glucans were developed for parenteral administration (see for example, [55–57]). These formulations underwent pre-clinical testing, and data have been published in the English literature (see for example [55, 58–60]). In non-clinical safety studies, mice, rats, guinea pigs and rabbits received a single i.v. injection of soluble beta-glucans in doses ranging from 40 to 1000 mg/kg [55]. Soluble beta-glucans administration did not induce mortality, change in appearance or behavioural changes in mice or rats. In subsequent studies, mice and guinea pigs were injected intraperitoneally (i.p.) with beta-glucans (250 mg/kg) for seven consecutive days. The mice gained weight at the same rate as the saline-treated controls. However, guinea pigs receiving i.p. injections of soluble beta-glucans showed a significant (*p* < 0.05) 10–13 % decrease in weight gain over the 7-day period. No other toxicological, behavioural or appearance changes were noted.

To examine chronic toxicity, soluble beta-glucans were administered to mice twice weekly for a period of 30 or 60 days, at doses of 40, 200 or 1000 mg/kg [55]. No deaths were observed in any group. Chronic beta-glucans administration did not alter body weight, liver, lung or kidney weight. However, significant splenomegaly was observed in both the 30 and 60-day studies. Histopathological examination showed no tissue alterations at 40 or 200 mg/kg. However, at 1000 mg/kg, a mononuclear infiltrate was observed in the liver.

Pyrogenicity testing in New Zealand white rabbits revealed that parenteral beta-glucans administration (5 mg/kg) did not significantly alter body temperature. The authors concluded that the systemic administration of soluble beta-glucans, over a wide dose range, does not induce mortality or significant toxicity in non-clinical studies [55].

## Beta-glucan levels in healthy subjects

Beta-glucans, probably of dietary origin, are detectable in the serum of healthy individuals. Normal levels have been determined as 17 pg/mL ± 34 pg/mL (0–51 pg/mL) [61] and 10–40 pg/mL [4]. Raised levels are a marker of invasive fungal infections, and commercially available assays for beta-glucans are used diagnostically, typically in immunocompromised patients [62, 63]. The serum of healthy humans also contains varying levels of anti-beta-glucan antibodies [64].

## Beta-glucan contamination of therapeutic products

False-positive results of assays for beta-glucans (i.e. true elevations in serum beta-glucan levels but not due to invasive fungal infections) have been described for patients given blood, blood derivatives or broad-spectrum antibiotics, in patients with severe mucositis or bacteraemia, and in patients undergoing major surgery or extracorporeal purification techniques such as haemodialysis, haemofiltration or haemodiafiltration (reviewed in [65]). In patients undergoing renal replacement therapy, the false positives are thought to be due to beta-glucans released from the filters and membranes used in dialysis. Serum levels up to 5561 pg/mL have been described following a single dialysis session using cellulose-containing membranes (reviewed in [65]).

Cellulose-containing filters used to process blood products are also thought to be the source of elevated beta-glucans levels in patients receiving blood products such as red blood cells, platelets or plasma products. Usami et al. detected beta-glucans contamination (defined as >20 pg/mL) in 75 % of albumin solutions, 40 % of blood coagulation factors and 63 % of immunoglobulin solutions tested [66]. Levels of beta-glucans as high as 7510 pg/mL were detected in these products. The authors estimated that plasma beta-glucans levels could reach 300 pg/mL after i.v. administration of 10 g of Gamma-Venin^®^ (a brand of immunoglobulin used in Japan and other countries), high enough to lead to an incorrect diagnosis of invasive fungal infection. The total dose of beta-glucans administered with a 10 g dose of Gamma-Venin^®^ was not provided. Of note, 10 g is within the range used for replacement of immunoglobulins in patients with hypogammaglobulinaemia (0.4–0.8 g/kg) but higher doses of immunoglobulin (1–2 g/kg, i.e. 70–140 g for a 70 kg adult) may be used in patients with diseases requiring immunomodulatory doses of immunoglobulins, such as immune thrombocytopenic purpura or Guillain–Barre syndrome.

In another study, high levels of beta-glucans were detected in the serum of patients 20 min after administration of various i.v. immunoglobulins and albumin [67]. The mean increases per 10 g of product ± standard deviation were as follows: Intractect^®^, 1632 ± 60 pg/mL; Privigen^®^, 300 ± 91 pg/mL; Octagam^®^, 194 ± 27 pg/mL; Kiovig^®^, 119 ± 22 pg/mL; and albumin 20 %, 156 ± 32 pg/mL. In a separate investigation, the authors also found what they described as “extraordinarily high” levels of beta-glucans in some patients who were recovering from Pneumocystis jirovecii infections (peak levels of 25,969 pg/mL, interquartile range 15,070–33,540 pg/mL) [67]. Since the patients were recovering from their illnesses, these findings could not be explained by the Pneumocystis jirovecii infection. As a result of this finding, the investigators considered that, although useful for diagnostic purposes, serum beta-glucans levels were not a reliable indicator of recovery in patients with Pneumocystis jirovecii infection.

Beta-glucans have also been described in antibody products in development for potential administration to humans, notably those produced in yeast cells or cultured with yeast-derived additives [68].

## Soluble beta-glucans as a therapeutic agent

Most recent publications relating to parenteral administration of soluble beta-glucans to humans have used lentinan, a form of beta-glucans extracted from shiitake mushrooms. Lentinan was approved in Japan in 1985 and in China in 1995, and it is widely used in these countries as an adjuvant to chemotherapy for patients with cancer. There appear to be at least six different formulations (of varying quality) available commercially (produced by five manufacturers) in China alone [69].

At least two other formulations of soluble beta-glucans have been developed for human use: a soluble beta-glucan from Biotec Pharmacon ASA, referred to simply as “SBG”, and Imprime PGG^®^ from Biothera Pharmaceuticals Inc. Trials of SBG appear to have completed recruitment but not been published (National Institutes of Health Clinical Trials identifiers NCT00533728 and NCT00533364). Imprime PGG^®^, also known as PGG-beta-glucan or poly-(1-6)**-**beta-glucotriosyl-(1-3)-beta-glucopyranose, is described as a soluble pharmaceutical grade, yeast-derived 1,3/1,6 beta-glucan.

Despite widespread use in China and Japan, there is relatively little clinical data on i.v. beta-glucans use published in the English literature. Key clinical studies of lentinan and Imprime PGG^®^ are summarised below.

### Lentinan

Most publications relating to the use of lentinan are in Japanese (usually with an English abstract), and these indicate that lentinan can be given via the i.v. (most commonly), intraperitoneal, intrapleural [70–72] and intraarterial routes [73]. The commonest dose/schedule appears to be 2 mg i.v. weekly, but i.v. doses as high as 10 mg have been given (see [6] for example). In addition, there are several ongoing Japanese phase II trials in patients with advanced gastric cancer (Trial identifiers in the World Health Organisation meta-register of clinical trials: JPRN-UMIN000010724, JPRN-UMIN000008590, JPRN-UMIN000007726 and JPRN-UMIN000001913). In these trials, where specified, lentinan is given i.v. at a dose of 2 mg weekly in combination with chemotherapy (TS-1, with or without cisplatin). A randomised phase III study of TS-1 alone versus TS-1 plus lentinan in advanced or recurrent gastric cancer appears to be complete but not published in the English literature. In this study, lentinan was given i.v. weekly but the dose is not specified.

The pharmacokinetics of lentinan given i.v. over 2 h at doses of 1, 2 or 4 mg, have been evaluated in healthy volunteers and gastric cancer patients [74]. After 1 mg of lentinan, plasma concentrations reached a maximum at the end of infusion (51–73 ng/mL) and decreased gradually thereafter. In healthy volunteers, lentinan concentrations 24 h after administration were (mean ± SD) 71 ± 21 ng/mL after a 4 mg dose, and 53 ± 11 ng/mL after a 2 mg dose. In three patients given 1 mg, levels were around 10–20 ng/mL after 24 h. Thereafter, levels declined slowly over approximately 7 days until they reached the detection limit. The pharmacokinetics of i.v. lentinan in humans were similar to those in the rat. Lentinan was found to be stable in human plasma, and the decline in plasma levels was thought to be due to uptake or degradation in cells, probably Kupffer cells in the liver.

In phase I/II placebo-controlled studies, HIV-positive patients were given 2, 5 or 10 mg of lentinan (or placebo) i.v. over 10 min once a week for 8 weeks, or 1 or 5 mg of lentinan i.v. over 30 min twice a week for 12 weeks [6]. Side effects were mainly mild, especially when the infusion was carried out over a 30-min period. When the infusion was over a 30-min period, there were no severe side effects and only four dropouts due to toxicity or patient preference. However, when administration was over a 10-min period, severe side effects occurred (one case each of anaphylactoid reaction, back pain, leg pain, depression, rigor, fever, chills, granulocytopenia and elevated liver enzymes) and four patients discontinued therapy as a result. Other investigators have also linked the side effects of lentinan (such as “oppression in the anterior chest” and dryness of the throat) to rapid infusions [7].

An individual patient-based meta-analysis of randomised trials of lentinan in gastric cancer included 650 patients from five trials of chemotherapy (combinations of S-1, mitomycin C and cisplatin), with or without lentinan (2 mg i.v. weekly) [75]. The concurrent use of lentinan with chemotherapy was found to significantly prolong overall survival compared with chemotherapy alone (hazard ratio 0.80; 95 % confidence intervals [CI] 0.68–0.95; stratified log rank *p* = 0.011). No major differences in haematological or non-haematological adverse events were reported for the two treatment regimens, although leucopenia was reported in 5.6 % of patients receiving chemotherapy plus lentinan versus 1.6 % of patients receiving chemotherapy alone. A meta-analysis has also been published of randomised chemotherapy trials, with or without lentinan, in non-small cell lung cancer (NSCLC) [76]. All the trials were conducted in China and published in Chinese. The addition of lentinan to chemotherapy resulted in higher response rates (relative risk [RR] = 1.31, 95 % CI 1.14–1.52) with less frequent Grade 3–4 gastrointestinal toxicity (RR = 0.54, 95 % CI 0.43–0.68) and Grade 3–4 granulocytopenia (RR = 0.65, 95 % CI 0.51–0.70) than with chemotherapy alone.

A randomised phase II trial published since the gastric cancer meta-analysis included 78 patients with advanced gastric cancer who received S1-based chemotherapy as first line treatment, with or without lentinan (2 mg i.v. every 2 or 3 weeks) [77]. Median overall survival was significantly longer in the lentinan group than in the chemotherapy alone group (689 days [95 % CI 431–2339 days] versus 565 days [95 % CI 323–662 days]; *p* = 0.0406). The most frequently observed severe (Grade 3–4) toxicity was neutropenia (chemo-immunotherapy 50 %, chemotherapy alone 45 %) but Grade 3 febrile neutropenia was only observed in one patient in each group. There were essentially no differences in the incidence or severity of adverse effects between patients who did and those who did not receive lentinan. Administration of lentinan was observed to suppress the granulocyte:lymphocyte ratio.

As described earlier, a 500 ng dose of beta-glucans could in theory be co-administered with a single dose of our product. In comparison, the maximum dose of i.v. lentinan used in routine practice or clinical trials is 10 mg, representing a safety margin of 1:20,000. From the study published by Yajima et al. [74], a 1 mg dose of lentinan given over 2 h resulted in a maximum plasma concentration of 73 ng/mL in patients with gastric cancer, indicating initial rapid clearance (since without any clearance the plasma concentration should have been around 200 ng/mL, based on a 5 L estimated blood volume). Extrapolating from these data suggests that a 500 ng dose of beta-glucans given over 2 h would only increase plasma levels to approximately 37 pg/mL, well within normal physiological levels of 17 pg/mL ± 34 pg/mL.

### Imprime PGG^®^

Imprime PGG^®^ has been evaluated in a range of clinical trials [78]. These indicate that Imprime PGG^®^ can be given i.v. at doses of 4 mg/kg weekly, and as single doses up to 6 mg/kg (for a 70 kg adult, this would equate to a 420 mg dose). Typical systemic clearance of beta-glucan in healthy subjects and cancer patients treated with Imprime PGG^®^ and cetuximab (with or without chemotherapy) were 0.491, 0.565, and 0.690 L/h, respectively [79]. The effective half-life of beta-glucan ranged from 19.5 to 27.3 h. In one study, mean area under the curve over 24 h of beta-glucan in Cycle 1 was similar to that in Cycle 3 (362 and 383 µg.hr/mL, respectively) [80]. Likewise, mean peak concentrations of beta-glucan in Cycles 1 and 3 were similar (44.3 and 47.8 µg/mL, respectively). Minimal accumulation of beta-glucan was observed with trough concentrations on Day 1 Cycle 2 and Day 1 Cycle 3 of 0.0680 and 0.117 μg/mL, respectively.

Detailed safety data are available from a randomised phase II study (*n* = 89) of paclitaxel, carboplatin and bevacizumab with or without Imprime PGG^®^ (4 mg/kg weekly) in patients with previously untreated NSCLC [81]. Events potentially associated with hypersensitivity reactions occurred more frequently with Imprime PGG^®^, including one Grade 3 anaphylactic reaction. As a result, the authors recommend premedication with low dose corticosteroids and anti-histamines prior to Imprime PGG^®^ administration. Of interest, overall and Grade 3–4 infections were less frequently reported in the Imprime PGG^®^ group compared to control (47.5 % vs 63.3 % overall; 5.1 % vs 10.0 % Grade 3–4). Immune-mediated adverse events (e.g. immune-mediated hepatitis or endocrinopathies) that are reported with T cell modulators (such as ipilimumab) were not observed with Imprime PGG^®^. Higher response rates, longer duration of response and improved survival were also reported for patients in the Imprime PGG^®^ group, although the study was not powered to demonstrate statistically significant improvements in these parameters.

Similar results were reported for a randomised phase II (*n* = 90) trial of Imprime PGG^®^ in combination with paclitaxel, carboplatin and cetuximab in NSCLC [82]. Overall (all grades) and Grade 3–4 adverse events potentially associated with hypersensitivity or infusion reactions were not increased in the Imprime PGG^®^ treatment arm in this study, but infections were less frequently reported (an absolute difference of 5 % between the two treatment groups). Immune-mediated adverse events (e.g. endocrinopathies) were not observed with Imprime PGG^®^. Higher response rates were reported in patients who received Imprime PGG^®^ in addition to standard therapy, especially in patients with higher levels of pre-existing anti-beta-glucans antibody levels, although time-to-event outcomes were balanced between the two groups.

Ongoing trials of Imprime PGG^®^ include a large (*n* = 795) randomised phase III trial in combination with cetuximab in patients with advanced *KRAS* wild-type colorectal cancer (NCT01309126). This trial started in April 2011 and is expected to complete in 2016. Other ongoing trials include a phase I/II trial of Imprime PGG^®^ in combination with rituximab in patients with indolent non-Hodgkin lymphoma (NCT02086175). A phase I/II trial of Imprime PGG^®^ in combination with an antibody and gemcitabine in pancreatic cancer was terminated early due to a drug recall (drug not specified) (NCT02132403).

The usual dose of Imprime PGG^®^ used in clinical trials is 4 mg/kg (280 mg for a 70 kg adult), 560,000 times higher than the maximum dose of beta-glucans (500 ng) that could be theoretically be administered with our product.

## Discussion and conclusions

Overall, potential administration of up to 500 ng of soluble beta-glucans as a contaminant of a biotherapeutic product is not considered a safety concern in view of the very much larger doses of soluble beta-glucans (lentinan, Imprime PGG^®^ and others) administered to humans i.v, the levels found in blood products and associated with dialysis, and reassuring preclinical studies. Both preclinical and clinical data indicate that beta-glucans are well tolerated, regardless of the route of administration. Doses as high as 4 mg/kg (approximately 560,000 times higher than 500 ng) have been repeatedly administered to humans i.v., without apparent ill-effects. Since biological agents such as monoclonal antibodies are generally administered by infusion over one to several hours, the chances of acute adverse effects due to beta-glucans contamination are further reduced; these effects appear to be mainly associated with rapid (10 min) infusions of lentinan (with doses of 1 mg or greater). Accordingly, a limit of 10 ng/mg (or 500 ng total dose) of beta-glucans is considered to pose a low risk to patients, and this specification was acceptable to the Medicines and Healthcare Products Regulatory Agency for our product. From a safety perspective, this level is probably considerably more stringent than necessary since it provides a very broad safety margin.

Much less is known about the levels at which the immunostimulatory effects of beta-glucans might occur. There is a dearth of clinical data in the English literature for doses lower than 2 mg/patient (the lowest dose commonly investigated in clinical efficacy trials of lentinan). However, in vitro studies suggest that significant immunostimulatory effects are unlikely to occur at serum beta-glucans concentrations lower than 1 ng/mL (1000 pg/mL), a concentration 10 times greater than the highest concentration likely to be encountered in our trial, especially with transient exposure. Most anti-tumour effects of beta-glucans appear to be mediated indirectly by its immunostimulatory properties. Although direct anti-proliferative and pro-apoptotic effects have been described, these appear to require higher concentrations of beta-glucans.

In patients with cancer (the intended patient population for the CRUKD/14/001 trial), possible immunostimulatory and/or direct anti-tumour effects of beta-glucan contaminants would, if anything, be considered desirable from a patient benefit perspective. However, in this first-in man, first-in-class, proof-of-concept trial, it is important to ensure that any anti-tumour efficacy observed is due to therapeutic MOv18 IgE itself. Moreover, for biotherapeutic agents developed for non-oncology indications, immunostimulatory effects would not necessarily be desirable. Based on currently available data, a limit of 10 ng/mg (or 500 ng total dose) of beta-glucans seems unlikely to provoke any clinically significant immunological effects and this level may be acceptable for medicinal agents.

## Abbreviations

CI: Confidence intervals
CR3: Complement receptor-3
CRUK: Cancer Research UK
IFN: Interferon
IκB: Inhibitor of κB kinase
IL: Interleukin
MIF: Migration inhibitory factor
NIHR: National Institute for Health Research
NSCLC: Non-small cell lung dancer
PGE2: Prostaglandin-E2
RR: Relative risk
TIMP: Tissue inhibitor of metalloproteinase
TME: Tumour microenvironment
